# Cognitive Functioning in Females with Endometriosis-Associated Chronic Pelvic Pain: A Literature Review

**DOI:** 10.1093/arclin/acaf003

**Published:** 2025-01-18

**Authors:** Ashlee Berryman, Liana Machado

**Affiliations:** Department of Psychology and Brain Health Research Centre, University of Otago, 275 Leith Walk, Dunedin North, Dunedin 9016, New Zealand; Department of Psychology and Brain Health Research Centre, University of Otago, 275 Leith Walk, Dunedin North, Dunedin 9016, New Zealand

**Keywords:** Endometriosis, Cognition, Cognitive performance, Neuropsychological, Chronic pain

## Abstract

**Objective:**

Cognitive complaints are commonplace among women living with endometriosis-associated chronic pelvic pain (CPP); yet, surprisingly few studies have assessed their cognitive functioning. As an initial step to address the resulting knowledge gap, this review aimed to synthesize the current literature reporting on cognition in females with endometriosis-associated CPP, and due to the poverty of endometriosis studies, to draw on data from female cohorts with other chronic pain conditions to gain potentially relevant insights.

**Methods:**

Three database searches were conducted (Scopus, PubMed, and PsycINFO). Eighteen studies met the inclusion criteria (*n* = 8 regarding endometriosis, *n* = 10 regarding other chronic pain conditions).

**Results:**

Six of the seven studies employing objective cognitive measures in females with other chronic pain reported significant cognitive deficits. Associated changes in neural circuitry thought to underpin these deficits align with neural patterns reported in females with endometriosis-associated CPP. While two studies reported a high prevalence (≥60%) of self-reported cognitive impairment in endometriosis-associated CPP, objective performance deficits have not been reported. Nonetheless, self-reported accounts of cognitive impairment suggest females with endometriosis-associated CPP may experience difficulties with inhibition, attention, and memory. Most studies did not control for potential moderating factors and comorbidities that prevail among endometriosis populations.

**Conclusion:**

The field is in desperate need of research assessing cognitive performance in females with endometriosis-associated CPP, to objectively determine any cognitive difficulties. Attention should be paid to likely moderators, pain-related mechanisms, and whether findings extend to endometriosis without CPP.

## INTRODUCTION

Pelvic endometriosis is a debilitating women’s health condition in which endometrial cells (endometriotic lesions) grow outside the uterus (Maulitz et al., 2022), commonly causing chronic pelvic pain (CPP)—noncyclic pain characterized as persisting for longer than 6 months (Howard et al., 2000). The disease affects over 190 million people of reproductive age with uteruses (Endometriosis New Zealand, 2023b), and is the leading cause of CPP in women globally (Szabo et al., 2022). Endometriotic lesions are characterized by an estrogen-dependent chronic inflammatory response over a menstrual period, involving the pelvic tissue, uterine tubes, ovaries, and in some cases nearby organs (namely the bowel and bladder; Bulun et al., 2019; Saunders & Horne, 2021). These lesions can cause cysts of the ovaries called endometriomas, and the surrounding organs/tissues may develop scars and sticky fibers (Bulun et al., 2019), further contributing to the CPP experienced by more than 50% of patients (Ballard et al., 2008; Sperschneider et al., 2019).

Pain is a highly subjective sensory perception and experience that, from an evolutionary perspective, functions to alert us to actual or potential tissue damage to drive protective behavioral changes (Eccleston & Crombez, 1999; Moriarty et al., 2011). However, when pain is treatment resistant, becomes chronic, or has an underlying pathology, it becomes maladaptive, which has diverse implications for both the sufferer and their support network (Hart et al., 2000; Howard et al., 2000). Historically, the biological, psychological, and physical ramifications of pain have been largely underestimated among the endometriosis population (Jones et al., 2004). In recent years it has been acknowledged that CPP appears to impose the largest negative influence on holistic wellbeing (Denny, 2004). Moreover, CPP irrespective of endometriosis is a robust predictor of cognitive functioning and poorer health-related quality of life (Tripp et al., 2004), which encompasses both objective pain and self-perceived health status (Karimi & Brazier, 2016).

To develop an informed understanding of why cognitive difficulties may be experienced by people suffering from endometriosis, it is important to first gain an understanding of the pathology and implications of endometriosis-associated CPP. Clinical evidence underscores the simultaneous presence of inflammatory, nociceptive, and neuropathic pain, with peripheral and central sensitization—which refers to the amplification of pain-related sensory input (Fleming & Volcheck, 2015)—playing important roles in the pain experience (Coxon et al., 2018; Maddern et al., 2020). The subsequent enhanced responsiveness and reduced threshold to pain, termed hyperalgesia, can result in abnormally enhanced pain sensations (Morotti et al., 2017). Further, common nerve pathways innervating organs in the pelvic region can result in cross-organ sensitization (Maddern et al., 2020), a process involving complex interactions between the nervous and immune system (Yong et al., 2020). This process offers a potential explanation for the variable pain experiences in endometriosis, which may at least in part determine the degree of cognitive disruptions. Moreover, endometriotic lesions have been found capable of developing their own nerve supply, establishing direct and bidirectional interactions with the central nervous system (Stratton & Berkley, 2011). This engagement enables direct participation of the hypothalamic–pituitary–adrenal axis, further contributing to diverse individual pain experiences (Morotti et al., 2017; Stratton & Berkley, 2011), and is therefore important to recognize when investigating pain networks in relation to cognitive functioning.

Several cross-sectional survey-based studies have revealed that endometriosis-associated pain intensity is a key predictor of experiencing negative affect (Jones et al., 2004), pessimism (Kumar et al., 2010), and alienation (Franz et al., 1986), all of which can heighten one’s pain perception and have downstream effects on cognitive functioning (Seminowicz & Davis, 2007). Hence, there may be a bidirectional relationship between pain cognition—the cognitive processing of pain perception (Khera & Rangasamy, 2021)—and neuropsychological functioning. In addition to influencing psychological parameters, numerous reviews have found endometriosis-associated pain can interfere with physical functioning (Hadi et al., 2019), ability to work (Endometriosis New Zealand, 2023b), interpersonal relationships (Jones et al., 2004), self-esteem (Kumar et al., 2010), and sleep (Stratton & Berkley, 2011), to name a few, most of which have been linked to cognitive functioning. These findings collectively signal a need for continued research efforts that address both the physiological *and* psychological aspects of pain processing and perception to improve our understanding of endometriosis-associated pain and its complex relationship with cognition before we can move toward improving cognitive outcomes.

At present, the only study to show objective cognitive deficits pertains to a primate model of endometriosis. In this study, 20 female common marmosets underwent testing with the Wisconsin General Test Apparatus, a device designed to investigate a wide range of cognitive abilities, including memory, problem-solving, and decision-making. Compared to healthy control marmosets, those with endometriosis (*n* = 12) showed a significantly slower learning rate (*p* = .006), and took longer to habituate to their environment (*p* = .008; Arnold et al., 2011). Together, these findings indicate that female marmosets with endometriosis experience impaired learning ability and long-term memory. In theory, such cognitive deficits should generalize to humans, considering the physiological and anatomical similarities between marmosets and humans (Arnold et al., 2011). However, endometriosis in animal models may not precisely reflect the pathophysiological and symptomatic aspects (such as CPP and sequelae) of endometriosis in humans, potentially limiting the applicability of these findings to the human endometriosis population.

Like many chronic pain conditions, women with endometriosis-associated CPP self-report impairments in cognition and daily functioning, which negatively affect work performance, social relationships, and quality of life (van Aken et al., 2017). It is therefore surprising and concerning that there is a notable lack of quantitative research exploring the relationship between CPP and cognitive functioning among the endometriosis population, despite at least 65% of people with endometriosis reporting some level of cognitive impairment (Fourquet et al., 2011). Hence, this review aimed to systematically analyze existing quantitative studies on cognitive functioning in women with endometriosis-associated CPP to better understand the nature of the cognitive difficulties experienced. Additionally, we sought to draw on knowledge stemming from research into other analogous female chronic pain cohorts (e.g., those with nociceptive and neuropathic pain conditions) due to potential for gaining valuable insights that could facilitate progress in endometriosis research.

## MATERIALS AND METHODS

This literature review was conducted with guidance from Preferred Reporting Items for Systematic Reviews and Meta-Analyses (PRISMA), an evidence-based set of guidelines for reporting systematic reviews (Moher et al., 2015). Database searches were conducted from May 2023 on Scopus, PubMed, and PsycINFO in accordance with recommendations from a librarian. Articles published between 1995 and 2024 were included in the review. Searches were conducted using predefined search strings combining terms related to endometriosis, cognition, and chronic pain using Boolean operators and truncation symbols where appropriate. The following search string was used for the endometriosis-specific search: (endometriosis) AND (cognition OR “cognitive performance” OR “cognitive function*” OR “executive function*” OR neuropsychological) AND (“chronic pain” OR “chronic pelvic pain” OR “pelvic pain”). Studies were only included if they fulfilled the following criteria: included biological females with any stage/classification of endometriosis (either laparoscopically/histologically confirmed or self-reported surgical diagnosis), who reported experiencing CPP, and included objective or self-reported cognitive measures.

**Fig. 1:**
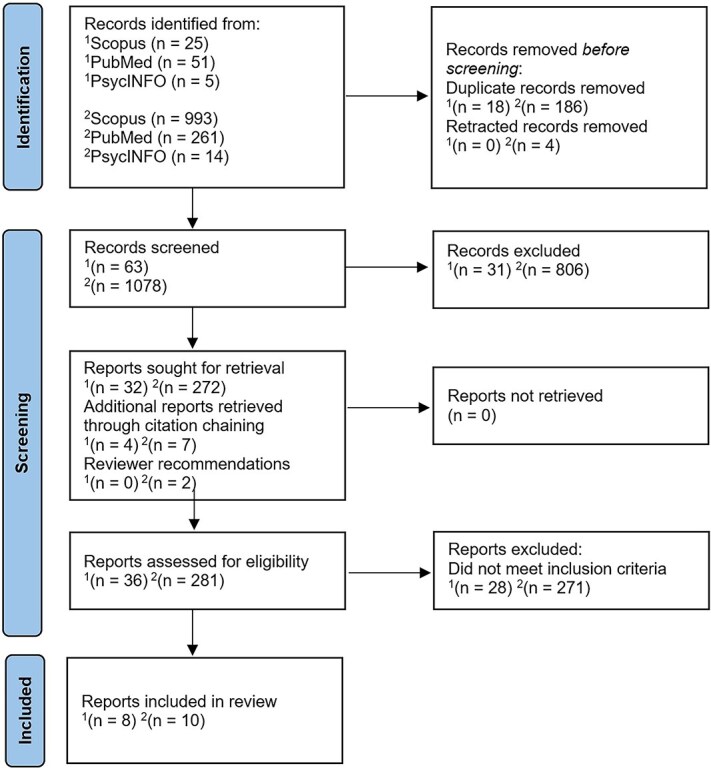
Flow diagram of systematic review adapted from “PRISMA 2020 flow diagram for new systematic reviews which included searches of databases and registers only”. Copyright 2023 by PRISMA. *Note.*  ^1^ = records from the endometriosis-specific search; ^2^ = records from other chronic pain search.

Given the scarcity of literature objectively exploring cognitive functioning in women with endometriosis-associated CPP, the following search string was used for the other chronic pain search: (female* OR women) AND (“neuropathic pain” OR “nociceptive pain” OR “pelvic pain” OR “abdominal pain” OR “lower back pain”) AND (cognition OR “cognitive performance” OR “cognitive function*” OR “executive function*” OR “neuropsychological”). Criteria for these studies were: biological females only (or separate statistical analyses run on biological females) reporting nociceptive or neuropathic pelvic, abdominal, or lower back pain, and included objective cognitive measures.

The first author screen titles and abstracts to determine potential eligibility, then reviewed full texts of potentially relevant articles. For both search strings, records that did not meet the stated criteria were excluded. No restrictions were applied regarding the country of publication. Risk of bias was not formally assessed due to limitations in the available literature and the heterogeneous nature of the included studies.

All articles from both search strings were transferred to Endnote reference manager and duplicates were removed. Additional reports were retrieved and included through secondary and tertiary citation chaining, providing they met the specified criteria. For both searches, the types of studies allowed were cohort studies, cross-sectional survey-based studies, case–control studies, experimental studies, meta-analyses of population-based studies, and review articles of clinical and preclinical research. See Fig. 1 for full study identification and screening procedures.

This review was not registered with any review registry. No deviations from the planned methodology were made.

**Table 1:** Overview of studies in females with endometriosis-associated CPP

Article	Design	Experimental group	Control group	Results
As-Sanie et al. (2012)	Cross-sectional study	Seventeen women with endometriosis-associated CPP, 15 women with endometriosis without CPP, and 6 women with CPP without endometriosis	Twenty-three age-matched healthy control women	Women with endometriosis and CPP showed decreased gray matter volume in the mid cingulate cortex, right putamen, and left thalamus. Women with CPP without endometriosis showed decreased gray matter volume in the left thalamus. Women with endometriosis without CPP showed decreased gray matter volume in the right inferior temporal gyrus and greater gray matter volume in the mesencephalon and the right prefrontal cortex (note *p* < .001 in all cases).
As-Sanie et al. (2016)	Cross-sectional study	Seventeen women with endometriosis-associated CPP and 13 women with endometriosis without CPP	Six age-matched women with CPP without endometriosis	Women with endometriosis-associated CPP showed enhanced glutamatergic neurotransmission and greater functional connectivity between the anterior insula and medial prefrontal cortex, and this connectivity was positively correlated with pain intensity (*r* = 0.55, *p* = .05).
Ferdek et al. (2019)	Cross-sectional study	Twenty women with endometriosis-associated CPP (*M_age_* = 35.6 years)	Seventeen healthy control women (*M_age_* = 34.6 years)	Women with endometriosis-associated CPP had greater resting connectivity from the left dorsolateral prefrontal cortex to the left somatosensory cortex (β = 0.24, *p* = .036) in the beta band (14–25 Hz) relative to healthy controls.
Fourquet et al. (2011)	Cross-sectional quantitative study	One hundred ninety-three women with self-reported laparoscopy confirmed endometriosis	-----	Work productivity survey: 60% ± 36.4 of women reported impairment in daily activities, 64% ± 35.5 reported a loss in level of work efficiency, and 65% ± 27.5 reported work impairment.
Sperschneider et al. (2019)	Multicenter case–control study	Five hundred five women with surgically /histologically confirmed endometriosis (*M_age_* = 37.5, *SD* = 7.3 years)	Five hundred five age-matched healthy control women (*M_age_* = 37.2, *SD* = 9.1 years)	A total of 89.8% of women with endometriosis reported a loss of productivity at work. CPP was significantly associated with this loss of productivity at work (OR = 3.08, *p* < .001), and the association was not influenced by the factor of endometriosis location (*p* > .05).
Steiner et al. (2020)	Cross-sectional study	Twenty women with endometriosis-associated CPP (*M_age_* = 28.5, *SD* = 5.2 years)	Twenty age-matched control women (*M_age_* = 28.5, *SD* = 5.2 years)	No significant behavioral differences were observed between the endometriosis and control groups during a continuous performance test (*p* = .094; Endometriosis [348.9 ± 64.1 ms]; Healthy controls [325.8 ± 51.9 ms]). Early P3a (*p* = .023), late P3a (*p* = .021), P3b (*p* = .024), and slow wave (*p* = .040) event-related potential amplitudes were significantly smaller for women with endometriosis compared to controls.
Szabo et al. (2022)	Pilot study	Eleven adolescent and young women with endometriosis-associated CPP (*M_age_* = 17.1, *SD* = 1.9)	Fourteen age-matched healthy female controls (*M_age_* = 16.6, *SD* = 2.7)	Adolescents and young women with endometriosis-associated CPP showed weaker functional connectivity between the right anterior insula and the middle frontal gyrus (*p* < .05), and between the right anterior insula and cerebellum (*p* < .05).
van Aken et al. (2017)	Cross-sectional questionnaire-based survey	Fifty women with laparoscopically and/or MRI proven endometriosis	Forty-two age-matched healthy control women	Women with endometriosis have significantly poorer health-related quality of life compared to healthy controls (*p* < .001). Pain intensity and pain cognition are independently negatively associated with health-related quality of life (Cohen’s *d =* 1.48, *p* < .001, and Cohen’s *d =* −1.53, *p* < .001, respectively).

*Note.* CPP = chronic pelvic pain; OR = odds ratio. The term “patients” indicates subjects participated in their capacity as patients. Missing age information indicates not reported.

## ENDOMETRIOSIS RESULTS

### Psychological factors of pain

Three of the eight studies in Table 1 exploring cognition in endometriosis samples are centered around cognitive intrusion due to pain, its subsequent psychological burden, and the influence of pain cognition. Based on the Medical Outcomes Study 36-item short-form health survey (Ware Jr. & Sherbourne, 1992) and the Endometriosis Health Profile 30 (Jones et al., 2001), van Aken et al. (2017) found that women with endometriosis-associated CPP (*n* = 50) are more likely than healthy controls (*n* = 42) to experience a significantly impaired quality of life (Cohen’s *d =* 1.48, *p* < .001, and Cohen’s *d =* −1.53, *p* < .001, respectively). Notably, both pain “intensity” and pain “cognition” had independent significant negative associations with health-related quality of life (van Aken et al., 2017). In a large cohort of 505 women with laparoscopy confirmed endometriosis, psychological symptoms were associated with greater perceived loss of work productivity (OR 2.90, 95% CI 1.98–4.23, *p* < .001; Sperschneider et al., 2019). Moreover, cognitive intrusion due to pain has been shown to substantially impact emotional health; Fourquet et al. (2011) found that among women with endometriosis-associated CPP (*n* = 193), 41% perceived that the pain controlled their life. Collectively, these studies establish that the psychological factors of pain associated with endometriosis are important determinants in one’s pain perception, and consequently quality of life.

### Functional and structural changes

The four neuroimaging studies identified through the endometriosis-specific search significantly contribute to our understanding of how psychological factors reinforce pain perception. For example, fMRI results have revealed that women with endometriosis-associated CPP have altered functional connectivity between the insular cortex and central pain networks, and these alterations are seen to a greater extent among women who exhibit the psychological factors of pain cognition (e.g., attention, expectancy, and appraisal; Peters, 2015; Szabo et al., 2022). In one pilot study of 11 adolescents and young women with endometriosis-associated CPP (10–24 years old, *M_age_* = 17.1, *SD* = 1.9), resting state fMRI revealed decreased functional connectivity between the right anterior insula and the middle frontal gyrus compared to age-matched healthy controls (13–21 years old, *M_age_* = 16.6, *SD* = 2.7; Szabo et al., 2022). Among healthy individuals, the middle frontal gyrus plays an important role in reorienting attention related to noxious stimuli (Japee et al., 2015), and the disruption of this circuitry is thought to contribute to the maintenance of enhanced pain states (Szabo et al., 2022). Decreased functional connectivity between the right anterior insula and the cerebellum was also observed (*p* < .05; Szabo et al., 2022); this circuitry is known to have a dominant inhibitory role in pain modulation (Moulton et al., 2010). Together, it appears the disruption of these modulatory circuits among women with endometriosis-associated pain is resulting in less effective top-down control of intrinsic pain modulation (e.g., attention allocation), contributing to the maintenance of enhanced pain experience, which may impact task-related attention (Seminowicz et al., 2011; Szabo et al., 2022).

This speculation is consistent with earlier fMRI research. Among women with endometriosis-associated CPP, enhanced neurotransmission and greater functional connectivity was observed between the anterior insula and medial prefrontal cortex (As-Sanie et al., 2016), key regions of the salience and default mode network. The salience network is a collection of cortical and subcortical regions, namely the anterior insula and dorsal anterior cingulate cortex, involved in determining allocation of attention (Uddin, 2016). Because of its functional role, the salience network serves as a “switch” between the default mode network and central executive network, collectively known as the Triple Network Model (Schimmelpfennig et al., 2023). The default mode network involves anterior and posterior cortical midline structures, thought to be activated during resting states and stimulus-independent thought (Giacino et al., 2014). Conversely, the central executive network encompasses lateral prefrontal regions and is activated during attentionally demanding cognitive tasks (Andrews-Hanna et al., 2014; Qin et al., 2015).

The default mode network and central executive network are often found to be anticorrelated, meaning that when one is active the other is inhibited (Fox et al., 2005). This anticorrelation allows for efficient switching between networks depending on cognitive demands required for a given task (Uddin et al., 2009). Interestingly, an enhanced connectivity between pain-related networks and the default mode network has been observed for women with endometriosis-associated CPP, but not for women with endometriosis without CPP, compared to healthy controls (As-Sanie et al., 2016). These observations suggest that women with endometriosis-associated CPP may have an impaired ability to disengage from heightened internal pain states, and therefore cognitively engage, which may affect performance on attentionally demanding tasks. This theory of disrupted disengagement is supported by the strength of the connectivity between the anterior insula and default mode network regions being positively correlated with pain intensity (*r = 0*.55, *p* = .05; As-Sanie et al., 2016). Similarly, neuroimaging studies have revealed functional abnormalities in brain regions linked to pain perception in women with dysmenorrhea (menstrual pain). Among 36 dysmenorrhea patients, greater amygdala functional connectivity in the default mode network and decreased amygdala functional connectivity in the ventral tegmental area (*p* < .005) was observed, together suggesting decreased pain modulation and poorer reward processing (Shen et al., 2019). These findings provide additional support for a relationship between pelvic pain and cognitive deficits.

Functional abnormalities relating to sensorimotor processing have also been observed in endometriosis. In one cross-sectional study utilizing electroencephalography, relative to healthy controls (*n* = 17), women with endometriosis-associated CPP (*n* = 20) had greater resting connectivity from the left dorsolateral prefrontal cortex to the left somatosensory cortex (β = 0.24, *p* = .036) in the beta band (14–25 Hz)—which represents movement preparation (Pani et al., 2014)—likely attributable to the continuous activation of the somatosensory pain system (Ferdek et al., 2019). In the same cohort, women with endometriosis-associated CPP exhibited greater connectivity from the left dorsolateral prefrontal cortex to the right temporal cortex (β = 0.34, *p* = .019) during pain-related imagery, potentially indicating compromised emotional regulation during the perception of pain-related stimuli. While these somatosensory changes may primarily manifest as impaired cognitive emotion regulation, altered functional connections induced by chronic pain have been shown to dysregulate a range of cognitive domains (Feller et al., 2020; Seo et al., 2012) and have consequently been theorized to cause impairment of higher-order cognitive performance among people with endometriosis (Ferdek et al., 2019).

Although functional connectivity changes in gynecological health conditions appear to be specific to those with CPP (As-Sanie et al., 2016; Shen et al., 2019), distinct brain volumetric changes have been found in both endometriosis with *and* without CPP. In one cross-sectional study, women who experienced CPP both with and without endometriosis (*n* = 17 and *n* = 6, respectively) showed decreased gray matter volume in pain-related brain regions compared to healthy controls (As-Sanie et al., 2012). Women with endometriosis without CPP did not demonstrate a decrease in gray matter volume in pain related regions but *did* show a decrease in the right inferior temporal gyrus (*p* < .001). This finding may suggest cognitive deficits for women with endometriosis without CPP, considering the role of the right inferior temporal gyrus in memory recall and visual perception (Onitsuka et al., 2004). Importantly, volumetric changes in the absence of CPP implies brain changes intrinsic to endometriosis, rather than solely manifesting from experienced pain. This underscores the complexity of neural mechanisms involved in endometriosis-related symptoms and signals likely (consequent) complexities in cognitive profiles.

### Self-reported and objective cognitive impairment

The modified functional connections associated with endometriosis may suggest impaired cognitive function, but direct links are not yet established. Nonetheless, there is evidence in isolation that endometriosis can adversely impact cognition, largely based on self-report. In a cross-sectional quantitative self-report study of 193 women with laparoscopy confirmed endometriosis, 60% reported experiencing impairment in daily activities, 64% reported experiencing a loss in level of work efficiency, and 65% reported experiencing work impairment (Fourquet et al., 2011). Similarly, a more recent matched case–control study of 505 women with surgically/histologically confirmed endometriosis found experiencing chronic pain was associated with impairment of professional life, demonstrated by a significant (self-estimated) loss of productivity at work (OR = 3.08, 95% CI 2.11–4.50, *p* < .001; Sperschneider et al., 2019). However, to our awareness, only one published study to date objectively assessed cognitive performance in individuals with endometriosis.

In a cross-sectional study of 20 women with moderate endometriosis-associated CPP (*M*  _pain severity_ = 4.1/10) and 20 age-matched healthy controls, event-related potentials and cognitive performance were measured during a cognitively demanding continuous performance task (the AX-CPT), which involves monitoring for an “A” followed by an “X”. Participants are instructed to respond quickly (without compromising accuracy) when they see the “X” after an “A”, allowing the assessment of visuomotor response, attention, and executive functions (Lopez-Garcia et al., 2016; Steiner et al., 2020). Early and late P3a, P3b, and slow wave event-related potential amplitudes were significantly smaller for women with endometriosis compared to controls, suggesting that endometriosis-associated CPP may cause changes in stimulus processing and inhibitory control networks. Regarding behavioral results, the direction of the data was consistent with poorer performance by the endometriosis group, however none of the performance metrics (reaction times, omission errors, and commission errors) statistically differed between women with endometriosis-associated CPP and healthy controls (*p* = .094, *p* = .125, and *p* = .127, respectively). Lack of statistical significance may at least in part reflect the small sample size, which suits detection of large effects only at 80% power.

As an alternative account for the contradiction between these results and studies reporting reduced executive functioning among other chronic pain samples, Steiner et al. (2020) proposed that compensatory neuroplasticity between areas of cognitive control and pain processing may have preserved cognitive performance. There may be merit to this theory, given the observed differences in functional connectivity among individuals with endometriosis-associated chronic pain between the anterior insula and middle frontal gyrus (Szabo et al., 2022). While the differences in functional connectivity between brain regions observed by Szabo et al. (2022) can be interpreted as indicating less neuronal transmission (Cansino, 2022), it could alternatively result from ongoing processing within compensatory brain regions not tapped by the cognitive tests assessed. Moreover, the greater functional connectivity observed between brain regions involved in pain processing and the medial prefrontal cortex in endometriosis patients implies increased engagement of anterior brain regions, typically associated with more attentionally demanding executive functions in healthy young adults (As-Sanie et al., 2016; Koechlin & Summerfield, 2007). The proposed compensatory neuroplasticity theory offers an explanation for the maintenance of behavioral performance, yet fails to address (i) why it would be the case that objective cognitive performance remains intact among women with endometriosis-associated CPP while it is generally impaired among other chronic pain conditions, and (ii) the high prevalence of self-reported cognitive impairment by women with endometriosis (Fourquet et al., 2011; Sperschneider et al., 2019).

Alternatively, the lack of significant differences in cognitive performance observed by Steiner et al. (2020) may be attributable to the specific cognitive task employed. The basic rule of the AX-CPT is relatively simple, requiring participants to sustain their attention to only a specific combination of letters (Lopez-Garcia et al., 2016). Earlier research has demonstrated that chronic pain patients exhibit objective performance deficits only when cognitive tasks are difficult and complex (Eccleston, 1994). The limitation of employing only one task fixed in level of difficulty fails to provide sufficient insight into the cognitive profile of women with endometriosis. This narrow approach may underpin the lack of significant objective cognitive deficits despite the extensive cognitive complaints voiced by people with endometriosis. Further exploration using a broader range of cognitive tasks with varying levels of complexity is warranted to better understand the nuanced cognitive impacts of endometriosis.

The three studies investigating self-reported and objective cognitive impairment did not consider participants’ experiences of cognitive intrusion due to pain and the impact on their task or work performance. Women with endometriosis who experience greater levels of cognitive intrusion due to CPP may potentially exhibit greater difficulties with task performance. Alternatively, this relationship may be more complex, as suggested by Attridge et al. (2015). For instance, efficient coping strategies may enable a subset of individuals with greater cognitive intrusion to mitigate this effect and maintain task performance. Given that cognitive intrusion from pain has been recognized as a crucial determinant of individual pain perception (van Aken et al., 2017)—where mental processes disrupted by pain affect the pain experience—subjective cognitive intrusion should be considered in future research.

## OTHER PAIN CONDITIONS RESULTS

Studies examining cognitive performance among women with chronic nociceptive and neuropathic pain conditions may offer valuable insights that can potentially translate to individuals with endometriosis-associated CPP. Generally speaking, women who suffer from chronic pain are more vulnerable to cognitive impairment than men (β = 0.12, *p* < .05; Roth et al., 2005). Among 222 chronic pain patients, female sex (*n* = 135) was significantly correlated with cognitive complaint items from the Brief Symptom Inventory of psychological functioning (*r* = 0.20, *p* < .05). This relationship may be mediated by heightened central sensitization and higher levels of pain catastrophizing among women, leading to fewer cognitive resources being available for executive functions (Roth et al., 2005; Smith Jr. et al., 2019). Hence, the association between chronic pain and cognition is intricate, at least when it comes to other chronic pain conditions.

### Objective cognitive impairment and functional changes

In one cohort study involving 386 Spanish female patients with chronic neuropathic pain and 491 with mixed neuropathic and nociceptive syndromes, the prevalence of cognitive impairment was assessed using the Mini Mental State Examination (Povedano et al., 2007). This standardized measure considers a score of ≤24 as indicative of cognitive impairment. Data revealed that women with chronic neuropathic pain had an observed prevalence of cognitive impairment at 23.0% (95% CI 18.1% - 28.0%), and women with mixed neuropathic and nociceptive syndromes had an observed prevalence at 18.9% (95% CI 14.6%–23.2%). These percentages considerably exceed the prevalence found in the Spanish population (1.0%–1.3%). Although some caution should be taken in making the comparison as the population data were not matched for sex and age, the adjusted prevalence of cognitive impairment remained markedly lower even after matching for sex and age.

**Table 2:** Overview of studies in females with other chronic neuropathic or nociceptive pain conditions

Article	Design	Experimental group	Control group	Results
Fernández-Palacios et al. (2024)	Case–control study	One hundred thirty women with fibromyalgia syndrome (*M_age_* = 54.67, *SD* = 9.43 years)	One hundred eleven age-matched healthy controls (*M_age_* = 55.31, *SD* = 13.97 years)	There was a significant main effect of group on neurocognitive test performance [Wilk’s λ = 0.874, *F*(19, 217) = 1.645, *p* = .048, η_p_^2^ = 0.126, β-1 = 0.942]. Women with fibromyalgia performed significantly worse than healthy controls on 11 out of 19 neurocognitive test measures (all *p* < .05, η_p_^2^ range = 0.001–0.043, mean η_p_^2^ = 0.014).
Glass et al. (2011)	Cross-sectional study	Eighteen females with fibromyalgia (*M_age_* = 43.6, *SD* = 9.79 years)	Fourteen age-matched healthy controls (*M_age_* = 41.13, *SD* = 11.91 years)	No significant differences were observed between fibromyalgia patients and healthy controls’ reaction times on a Go/No-Go task (577.50 versus 570.63 ms, respectively, *F*(1, 30) = 0.117, *p* = .735).
Landrø et al. (1997)	Multidimensional cross-sectional study	Twenty-five females with primary fibromyalgia (*M_age_* = 46.4, *SD* = 10.4 years)	Eighteen healthy (not matched) controls (*M_age_* = 40.1, *SD* = 9.6 years)	Fibromyalgia patients performed significantly worse than healthy controls on the Randt Memory Test [*F*(1, 40) = 6.1, *p* < .05], a word fluency task [*F*(1, 40) = 5.5, *p* < .05], the Code Memory Test Part 1 [*F*(1, 40) = 7.6, *p* < .01], and the Code Memory Test Part 2 [*F*(1, 40) = 8.6, *p* < .01].
Martucci et al. (2015)	Cross-sectional study	Forty-five female patients with urologic CPP syndrome (*M_age_* = 40.3 years)	Forty-five healthy female controls (*M_age_* = 38.1 years)	Urologic CPP syndrome patients exhibited decreased functional connectivity of the default mode network to the posterior cingulate cortex and left precuneus, and greater connectivity between the posterior cingulate cortex and brain regions implicated in pain and somatosensory regulation processes (*p* < .05).
Naliboff et al. (2015)	Cross-sectional case–control study	Two hundred thirty-three female patients with urologic CPP syndrome (*M_age_* = 40.5 years)	Two hundred thirty-five age-matched healthy female controls (*M_age_* = 38.1 years)	Ability Self-Report Questionnaire revealed urological CPP syndrome patients showed significantly poorer performance in language (β_std_ = 0.65, CI 1.71–3.63), visual perceptual ability (β_std_ = 0.50, CI 0.93–2.56), verbal memory (β_std_ = 0.55, CI 1.5–3.78), and visual spatial memory (β_std_ = 0.42, CI 0.71–2.58).
Povedano et al. (2007)	Pragmatic cohort-study	Three hundred eighty-six female patients with chronic neuropathic pain (*M_age_* = 58.4, *SD* = 14.4 years), 491 with mixed neuropathic and nociceptive syndromes (*M_age_* = 55.1, *SD* = 12.9 years)	The Spanish population	Mini-Mental State Examination observed prevalence of cognitive impairment for neuropathic pain at 23.0% (95% CI 18.1% - 28.0%) and observed prevalence of cognitive impairment for mixed neuropathic and nociceptive syndromes at 18.9% (95% CI 14.6%–23.2%), which both considerably exceeded the prevalence found in the Spanish population (1%–1.3%).
Roth et al. (2005)	Cross-sectional survey-based study	One hundred thirty-five women and 87 men with chronic pain conditions (*M_age_* = 49.8, *SD* = 9.6 years)	—	Female sex had a significant contribution to the report of cognitive complaints (β = 0.12, *p* < .05), whereby female sex was significantly correlated with more total cognitive complaints (*r* = 0.20, *p* < .05).
Serrano et al. (2022)	Exploratory cross-sectional study	Sixty-nine female fibromyalgia patients (*M_age_* = 49.04, *SD* = 9.39 years)	Twenty-one healthy female controls (*M_age_* = 45.67, *SD* = 10.79 years)	Fibromyalgia patients scored significantly lower than healthy controls on the forward digit span (β = −3.572, *p* = 0.021), and the Controlled Oral Word Association Test (β = −16.451, *p* = .021).
Shen et al. (2019)	Cross-sectional study	Thirty-six female primary dysmenorrhea patients (*M_age_* = 22.36, *SD* = 2.95 years)	Thirty-five age-matched healthy female controls (*M_age_* = 22.97, *SD* = 1.76 years)	Primary dysmenorrhea patients exhibited greater amygdala functional connectivity in the default mode network, and decreased amygdala functional connectivity in the ventral tegmental area (*p* < .005) compared to healthy controls, suggesting decreased pain modulation and poorer reward processing.
Verdejo-García et al. (2009)	Cross-sectional study	Thirty-six women diagnosed with fibromyalgia (*M_age_* = 45.86, *SD* = 6.78 years)	Thirty-six healthy age-, education-, and socioeconomic status-matched controls (*M_age_* = 44.97, *SD* = 6.70 years)	Women with fibromyalgia showed a flat learning curve in the Iowa Gambling Task relative to healthy controls: Control [*F*(4,33) = 2.58, *p* < .05]; Fibromyalgia [*F*(4,33) = 0.69, *p* > .05], greater mean non-perseverative errors on the Wisconsin Card Sorting Test than healthy controls (46.71 versus 40.72, respectively, *p* = .04), and significantly lower mean number of categories (3.08 versus 4.36, respectively, *p* = .007).

*Note.* CPP = chronic pelvic pain; CI = 95% confidence interval; β_std_ effect size measure = β divided by the variable’s standard deviation.

In a cross-sectional case–control study, 233 women with urologic CPP syndrome (UCPPS) and 235 sex- and education-matched healthy controls underwent a range of Multiple Ability Self-Report Questionnaires (Naliboff et al., 2015). Language (β_std_ = 0.65, 95% CI 1.71–3.63), visual perceptual ability (β_std_ = 0.50, 95% CI 0.93–2.56), verbal memory (β_std_ = 0.55, 95% CI 1.5–3.78), and visual spatial memory scores (β_std_ = 0.42, 95% CI 0.71–2.58) were significantly lower for women with UCPPS compared to healthy controls (*p* < .01). Further, although not statistically significant, there was a large effect of attentional concentration between UCPPS patients and healthy controls (β_std_ = 0.57, 95% CI 1.54–3.86, *p* > .01). These self-reported cognitive complaints by women with UCPSS match the earlier objective performance deficits seen by Povedano et al. (2007), and align with the self-reported cognitive complaints of endometriosis patients (Fourquet et al., 2011; Sperschneider et al., 2019). Moreover, fMRI data from female UCPPS patients indicate greater functional connectivity among brain regions associated with pain, sensory, and motor processing (Martucci et al., 2015). Conversely, the left precuneus—a key region of the default mode network—displayed weaker functional connectivity to prefrontal and parietal regions involved in reward processing and executive functioning (Martucci et al., 2015). Together, these findings suggest a relationship between altered brain activity and objective cognitive deficits among chronic pain patients.

Insights can also be gleaned from various nonpelvic pain conditions, such as fibromyalgia, which shares the symptom of idiopathic neuropathic pain with endometriosis (Lamvu et al., 2021). In a multidimensional cross-sectional study, 25 female patients with fibromyalgia and 18 healthy female controls underwent a range of cognitive and memory tasks that varied in difficulty (Landrø et al., 1997). Data revealed fibromyalgia patients performed significantly worse than healthy controls on a range of executive functions, including working memory, verbal planning capacity, and memory coding (refer to Table 2 for statistical details). Analogous results emerged in two subsequent cross-sectional studies and one case–control study among women with fibromyalgia. In the first cross-sectional study, women with fibromyalgia exhibited impaired abstract reasoning and mental set shifting; in addition, they displayed a flat learning curve in the Iowa Gambling Task compared to healthy controls (Verdejo-García et al., 2009). In the other cross-sectional study, female fibromyalgia patients showed impaired short-term verbal and working memory, and verbal fluency (Serrano et al., 2022), although it should be acknowledged that the 3-year mean age discrepancy between the fibromyalgia patients and controls was not in the conservative direction.

In the case–control study, women with fibromyalgia showed significantly poorer performance compared to healthy controls on 11 out of 19 neurocognitive test measures (all *p* < 0.05; Fernández-Palacios et al., 2024). Whereas no significant performance differences emerged for the executive functions working memory and planning, deficits were observed in selective attention, long-term visual memory, and processing speed, with the most pronounced executive-level challenge being in inhibitory control, potentially aligning with self-reported work-related difficulties described by women with endometriosis (Fourquet et al., 2011; Sperschneider et al., 2019). In contrast, another study observed no differences in reaction times between fibromyalgia patients and healthy controls on a Go/No-Go task [577.50 versus 570.63 ms, respectively, *F*(1, 30) = 0.117, *p* = .735; Glass et al., 2011], a well-established paradigm for assessing inhibitory response (Rezvanfard et al., 2016). These conflicting results seem to align with theories proposed by Eccleston (1994), suggesting that cognitive deficits may manifest only when tasks meet a certain level of difficulty, which aligns with our suggestion that the continuous performance task utilized by Steiner et al. (2020) may have been too simplistic to detect significant group differences among women with endometriosis-associated CPP.

## DISCUSSION

This review found that chronic pain has been linked to both functional and structural changes in brain regions associated with cognitive control, emotional processing, and nociception, and that objective cognitive deficits among chronic pain populations were seen in most of the relevant studies. These deficits were particularly pronounced in tasks that require shifting between the default mode network and the salience network (Martucci et al., 2015; Shen et al., 2019). However, there is a lack of research investigating objectively measured cognitive difficulties among women with endometriosis-associated CPP. This gap in research may be explained by the priority to develop acute non-invasive diagnostic methods and potential treatments for endometriosis (Rogers et al., 2017). Moreover, underestimation of the prevalence of endometriosis may further deprioritize cognitive-based research, as current population statistics only include individuals with laparoscopy-confirmed endometriosis (Endometriosis New Zealand, 2023a). Consequently, the true number of women who may experience endometriosis and subsequent cognitive impairments (potentially with or without CPP) is likely greater than what current research suggests.

Women with endometriosis-associated CPP exhibited changes in both functional and structural brain connectivity, resembling those observed in individuals with other chronic pain conditions, who exhibit objective cognitive deficits. While these altered neural connections have only been found for endometriosis with CPP (As-Sanie et al., 2016), distinct volumetric changes have been reported for endometriosis without CPP (As-Sanie et al., 2012). Research to date suggests the most likely candidate for influencing cognition is the component chronic pain, particularly given the evidence of endometriotic lesions developing their own nerve supply directly to the central nervous system (Stratton & Berkley, 2011) and the frequency of self-reported cognitive impairment in the presence of CPP (Fourquet et al., 2011; Sperschneider et al., 2019); however, this does not preclude the possibility of cognitive dysfunction in endometriosis without CPP.

**Fig. 2:**
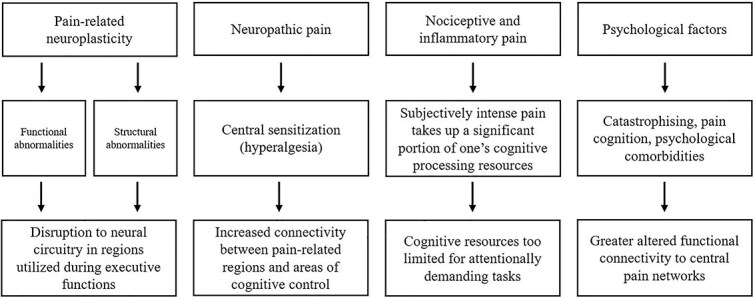
Schematic of four potential pathways for cognitive distinctions in women with endometriosis-associated CPP. *Note.* Although the pathways are presented separately, substantial interactions exist.

Drawing on the findings pertaining to endometriosis and other chronic pain conditions, four potential pathways currently appear to underlie the cognitive distinctions that may arise in women with endometriosis experiencing CPP (outlined in Fig. 2). Pain-related neuroplasticity involving either functional or structural brain changes can disrupt executive functions (As-Sanie et al., 2012; As-Sanie et al., 2016), while central sensitization in neuropathic pain heightens connectivity between pain and cognitive control networks (Shen et al., 2019), potentially amplifying cognitive disruptions. Nociceptive and inflammatory pain may further deplete cognitive resources, whereby the processing of pain is (typically) innately prioritized over the demands of executive functioning (Ahmad & Aziz, 2014). Psychological factors arising from prolonged noxious input, such as in in CPP, can reciprocally alter functional connectivity (Price et al., 2006), again potentially exacerbating cognitive deficits. It is noteworthy that while the pathways are presented independently, there are substantial interactions. Together, these interconnected mechanisms provide a framework for understanding cognitive disruptions in women with endometriosis-associated CPP.

Based on the studies identified through the other chronic pain conditions search, chronic pain was in most cases associated with cognitive impairment—which may be attributed to the fact that pain consumes a substantial portion of our limited cognitive resources (Phelps et al., 2021)—because chronic pain and cognitive processing utilize overlapping neural circuits in the central nervous system (Glass et al., 2011). One issue that remains is the difficulty in determining whether cognitive performance is preserved in endometriosis, as implied by Steiner et al. (2020), and if not whether objective cognitive deficits are dependent on individual pain intensity. Among chronic pain populations, it appears subjective pain intensity is a large predictor of poorer cognitive performance. Not only did Eccleston (1994) find that performance deficits could be seen only when cognitive tasks were difficult and complex, but also the extent of those deficits depended on individual perceived pain intensity (Eccleston, 1995); in an attentionally demanding interference task, mean reaction times were significantly slower for patients with high pain intensity compared to patients with low pain intensity (*p* < .05) and pain-free healthy controls (*p* < .05).

Yet, when it comes to women with endometriosis-associated CPP, no clear evidence of objective performance deficits emerged, despite the participants having moderate pain severity (Steiner et al., 2020). As mentioned earlier, this comes as a surprise given the frequency of self-reported claims of cognitive impairment interfering with daily functioning. Given the absence of effect size reporting in studies on self-reported cognitive complaints in endometriosis, no comment can be made regarding the magnitude of the effects. However, it is noteworthy that the percentage of women with endometriosis-associated CPP who self-reported cognitive impairments (resulting in decreased work productivity and efficiency) was high across the two survey-based studies, with ranges of 60%–65% (Fourquet et al., 2011), and 65.1%–89.8% (Sperschneider et al., 2019).

Existing pain psychology frameworks can help us understand the subjective cognitive disruptions in women with endometriosis. According to the gate control theory of pain, pain perception is subject to modulation by both peripheral input and central mechanisms (Melzack & Wall, 1965). This could explain why some cognitive tasks—namely those that activate central inhibitory processes—can reduce pain perception. For instance, a recent experimental study demonstrated that perceived pain intensity from an experimentally induced pain stimulus was significantly lower during 1-back and 2-back working memory tasks (*p* < .001) compared to baseline (Tejera-Alonso et al., 2024). Notably, this effect was consistent across individuals regardless of their cognitive abilities or attention levels. The biopsychosocial model, on the other hand, emphasizes the interrelationship between biological, psychological, and social factors in pain perception (Engel, 1977), aligning with findings of cognitive complaints, work-related challenges, and reduced quality of life reported in endometriosis (Fourquet et al., 2011; Sperschneider et al., 2019; van Aken et al., 2017). Similarly, the pain matrix theory highlights the role of multiple interconnected brain networks in pain processing and perception (Melzack, 2001), including cognitive and emotional regulation systems (Glass et al., 2011). The more comprehensive central sensitization theory integrates these perspectives, describing how chronic pain states are associated with heightened central nervous system responsiveness and sequelae symptomatology, as observed in endometriosis.

### Limitations and future directions

Several considerations for future research have stemmed from this review that have not yet been addressed. Firstly, none of the studies offered a clear definition of CPP to participants, nor did they differentiate it from dysmenorrhea (menstrual pain). According to a meta-analysis of population-based studies, there is a positive correlation between dysmenorrhea and both the presence and severity of CPP (irrespective of an endometriosis diagnosis). Among 6,689 women from 8 studies, those with CPP had 2.43 times the odds of experiencing dysmenorrhea compared to those without CPP (95% CI 1.98–2.99, *p* < .00001), and these odds were consistent across population types and geographical regions (Li et al., 2020). Thus, although CPP and dysmenorrhea are distinct in etiology, they frequently co-occur. In fact, a recent cross-sectional study of 527 women with endometriosis revealed that *all* reported experiencing dysmenorrhea (albeit to varying degrees; Markham et al., 2019), whereas the rate of dysmenorrhea is between 16.8% and 81% in the general population (Latthe et al., 2006; discussed in MacGregor et al., 2023). Further, systematic reviews have suggested that dysmenorrhea may predispose women to CPP by enhancing central sensitization (Li et al., 2020; Li et al., 2023)—the amplification of pain-related sensory input (Fleming & Volcheck, 2015)—which has been shown to negatively impact cognitive performance (Serrano et al., 2022). It is therefore important that a clear differentiation between dysmenorrhea and CPP is established in studies of pain among women with endometriosis, especially when assessing self-reported subjective pain ratings, rather than utilizing objective pain measures.

Secondly, moderators of cognitive functioning were seldom controlled for in studies investigating cognitive performance. While some studies included control groups that were age- and education-matched, factors such as medication usage, physical activity, sleep quality, and psychological comorbidities were rarely considered. Regarding the latter, although CPP appears to be a significant predictor of self-reported cognitive difficulties, it is important to acknowledge that cognitive dysfunction may also be influenced by psychological and non-pain comorbidities associated with endometriosis. For instance, cognitive challenges associated with endometriosis may be exacerbated by factors like anxiety and depression (Roth et al., 2005; Sepulcri & do Amaral, 2009). It is imperative to address these variables in future research involving the endometriosis population to gain a more comprehensive understanding of the intricate relationship between endometriosis-associated CPP and cognition. Unpacking the complex intricacies may ultimately lead to better outcomes for those affected by this condition.

The main limitation of this review is the poverty of research on cognition among endometriosis cohorts. While it is evident that there is a scarcity of research in this area, it could be that we failed to find some existing studies. Despite employing comprehensive search strategies with advice from an experienced librarian and retrieving additional reports via citation chaining, the set search strings and inclusion criteria may have resulted in relevant studies being missed, leading to incomplete evidence synthesis. Future research endeavors should therefore focus on expanding search strategies and inclusion criteria to ensure a more comprehensive and exhaustive review of relevant literature, thereby enhancing the robustness and reliability of evidence synthesis. Such methodological considerations may include expanding the search string to include inflammatory pain and cyclical (non-chronic) pain profiles, as well as expanding criteria to encompass other common pain disorders associated with endometriosis (e.g., secondary dysmenorrhea, vaginismus, inflammatory bowel disease, back pain).

Finally, population characteristics, particularly sample sizes and ethnicity, as well as outcome measures differed across studies; the high level of heterogeneity may limit the generalizability and application of conclusions drawn in this review. To address this limitation, future studies should strive for more standardized methodologies, including consistent outcome measures and statistical methods, to facilitate more accurate comparisons and enhance the relevance of findings to broader endometriosis cohorts. Researchers should also strive to achieve samples with ethnic diversity that accurately mirrors the demographics of the population.

### Additional considerations

The presence of objective cognitive deficits in other chronic pain conditions may suggest that the self-reported cognitive complaints in endometriosis result from CPP. While all the studies in this review indicate that women with endometriosis who experience cognitive impairment also experience CPP, as mentioned earlier, it remains plausible that cognitive deficits may exist in women with endometriosis even in the absence of CPP, given the evidence of differences in brain volume and various psychological and non-pain comorbidities-associated with the condition. However, based on significant large effects of impaired executive functioning observed in five chronic pain studies, it is reasonable to assume that the presence of CPP and its associated psychological factors may have a more pronounced impact on cognitive performance compared to endometriosis without CPP. Nevertheless, the inclusion of a distinct group of women with endometriosis who do not have CPP is imperative for a better understanding of the intricate relationship between endometriosis (and its many non-pain comorbidities) and cognitive function.

## CONCLUSION

Overall, this review indicates that women with endometriosis-associated CPP report inefficiencies in cognitive functioning, largely inhibition, attention, and memory. However, objectively measured cognitive deficits are yet to be seen (at least in humans). There is a need for future quantitative research to gain a more comprehensive understanding of the cognitive profiles of women with endometriosis-associated CPP, to provide crucial information regarding the underexplored ramifications of the condition that may inform treatment strategies, and to help people living with endometriosis-associated CPP better understand their situation.

## AVAILABILITY OF DATA AND MATERIALS

Correspondence concerning this article should be addressed to Ashlee Berryman, Department of Psychology, University of Otago, William James Building, 275 Leith Walk, Dunedin, 9054, New Zealand. Email: ashlee.berryman@otago.ac.nz

## References

[ref1] Ahmad, A. H., & Aziz, C. B. A. (2014). The brain in pain. The Malaysian Journal of Medical Sciences: MJMS, 13(4), 48. 10.1097/01.bonej.0000265758.09846.32. PMC4405805 25941463

[ref2] van Aken, M. A., Oosterman, J. M., Van Rijn, C., Ferdek, M. A., Ruigt, G. S., Peeters, B., et al. (2017). Pain cognition versus pain intensity in patients with endometriosis: Toward personalized treatment. Fertility and Sterility, 108(4), 679–686. 10.1016/j.fertnstert.2017.07.016. 28911933

[ref3] Andrews-Hanna, J. R., Smallwood, J., & Spreng, R. N. (2014). The default network and self-generated thought: Component processes, dynamic control, and clinical relevance. Annals of the New York Academy of Sciences, 1316(1), 29–52. 10.1111/nyas.12360. 24502540 PMC4039623

[ref4] Arnold, C., Lamp, J., Lamp, O., & Einspanier, A. (2011). Behavioral tests as indicator for pain and distress in a primate endometriosis model. Journal of Medical Primatology, 40(5), 317–326. 10.1111/j.1600-0684.2011.00496.x. 21950721

[ref5] As-Sanie, S., Harris, R. E., Napadow, V., Kim, J., Neshewat, G., Kairys, A., et al. (2012). Changes in regional gray matter volume in women with chronic pelvic pain: A voxel-based morphometry study. PAIN®, 153(5), 1006–1014. 10.1016/j.pain.2012.01.032. 22387096 PMC3613137

[ref6] As-Sanie, S., Kim, J., Schmidt-Wilcke, T., Sundgren, P. C., Clauw, D. J., Napadow, V., et al. (2016). Functional connectivity is associated with altered brain chemistry in women with endometriosis-associated chronic pelvic pain. Journal of Pain, 17(1), 1–13. 10.1016/j.jpain.2015.09.008. 26456676 PMC4698023

[ref7] Attridge, N., Crombez, G., Van Ryckeghem, D., Keogh, E., & Eccleston, C. (2015). The experience of cognitive intrusion of pain: Scale development and validation. Pain, 156(10), 1978–1990. 10.1097/j.pain.0000000000000257. 26067388 PMC4770387

[ref8] Ballard, K., Seaman, H., De Vries, C. S., & Wright, J. (2008). Can symptomatology help in the diagnosis of endometriosis? Findings from a national case–control study—Part 1. BJOG: An International Journal of Obstetrics and Gynaecology, 115(11), 1382–1391. 10.1111/j.1471-0528.2008.01878.x. 18715240

[ref9] Bulun, S. E., Yilmaz, B. D., Sison, C., Miyazaki, K., Bernardi, L., Liu, S., et al. (2019). Endometriosis. Endocrine Reviews, 40(4), 1048–1079. 10.1210/er.2018-00242. 30994890 PMC6693056

[ref10] Cansino, S. (2022). Brain connectivity changes associated with episodic recollection decline in aging: A review of fMRI studies. Frontiers in Aging Neuroscience, 14, 1225. 10.3389/fnagi.2022.1012870. PMC9640923 36389073

[ref11] Coxon, L., Horne, A. W., & Vincent, K. (2018). Pathophysiology of endometriosis-associated pain: A review of pelvic and central nervous system mechanisms. Best Practice & Research Clinical Obstetrics & Gynaecology, 51, 53–67. 10.1016/j.bpobgyn.2018.01.014. 29525437

[ref12] Denny, E. (2004). Women’s experience of endometriosis. Journal of Advanced Nursing, 46(6), 641–648. 10.1111/j.1365-2648.2004.03055.x. 15154905

[ref13] Eccleston, C. (1994). Chronic pain and attention: A cognitive approach. British Journal of Clinical Psychology, 33(4), 535–547. 10.1111/j.2044-8260.1994.tb01150.x. 7874045

[ref14] Eccleston, C. (1995). Chronic pain and distraction: An experimental investigation into the role of sustained and shifting attention in the processing of chronic persistent pain. Behaviour Research and Therapy, 33(4), 391–405. 10.1016/0005-7967(94)00057-Q. 7538753

[ref15] Eccleston, C., & Crombez, G. (1999). Pain demands attention: A cognitive–affective model of the interruptive function of pain. Psychological Bulletin, 125(3), 356–366. 10.1037/0033-2909.125.3.356. 10349356

[ref16] Endometriosis New Zealand (2023a). Diagnosis of endometriosis. Endometriosis New Zealand. https://nzendo.org.nz/endo-information/#diagnosis.

[ref17] Endometriosis New Zealand (2023b). Submission to Manatū Hauora Ministry of Health on the Aotearoa New Zealand Women’s Health Strategy. New Zealand: Ministry of Health. https://nzendo.org.nz/wp-content/uploads/2023/03/Endometriosis-New-Zealand-Submission_1-1.pdf.

[ref18] Engel, G. L. (1977). The need for a new medical model: A challenge for biomedicine. Science, 196(4286), 129–136. 10.1126/science.847460. 847460

[ref19] Feller, L., Feller, G., Ballyram, T., Chandran, R., Lemmer, J., & Khammissa, R. A. G. (2020). Interrelations between pain, stress and executive functioning. British Journal of Pain, 14(3), 188–194. 10.1177/2049463719889380. 32922780 PMC7453485

[ref20] Ferdek, M. A., Oosterman, J. M., Adamczyk, A. K., van Aken, M., Woudsma, K. J., Peeters, B. W., et al. (2019). Effective connectivity of beta oscillations in endometriosis-related chronic pain during rest and pain-related mental imagery. Journal of Pain, 20(12), 1446–1458. 10.1016/j.jpain.2019.05.011. 31152855

[ref21] Fernández-Palacios, F. G., Pacho-Hernández, J. C., Fernández-de-Las-Peñas, C., Gómez-Calero, C., & Cigarán-Méndez, M. (2024). Evaluation of cognitive performance in patients with fibromyalgia syndrome: A case–control study. Life, 14(5), 649. 10.3390/life14050649. 38792669 PMC11122595

[ref22] Fleming, K. C., & Volcheck, M. M. (2015). Central sensitization syndrome and the initial evaluation of a patient with fibromyalgia: A review. Rambam Maimonides Medical Journal, 6(2), e0020. 10.5041/RMMJ.10204. 25973272 PMC4422459

[ref23] Fourquet, J., Báez, L., Figueroa, M., Iriarte, R. I., & Flores, I. (2011). Quantification of the impact of endometriosis symptoms on health-related quality of life and work productivity. Fertility and Sterility, 96(1), 107–112. 10.1016/j.fertnstert.2011.04.095. 21621771 PMC3129383

[ref24] Fox, M. D., Snyder, A. Z., Vincent, J. L., Corbetta, M., Van Essen, D. C., & Raichle, M. E. (2005). The human brain is intrinsically organized into dynamic, anticorrelated functional networks. Proceedings of the National Academy of Sciences, 102(27), 9673–9678. 10.1073/pnas.0504136102. PMC1157105 15976020

[ref25] Franz, C., Paul, R., Bautz, M., Choroba, B., & Hildebrandt, J. (1986). Psychosomatic aspects of chronic pain: A new way of description based on MMPI item analysis. Pain, 26(1), 33–43. 10.1016/0304-3959(86)90171-5. 2942831

[ref26] Giacino, J. T., Fins, J. J., Laureys, S., & Schiff, N. D. (2014). Disorders of consciousness after acquired brain injury: The state of the science. Nature Reviews Neurology, 10(2), 99–114. 10.1038/nrneurol.2013.279. 24468878

[ref27] Glass, J. M., Williams, D. A., Fernandez-Sanchez, M.-L., Kairys, A., Barjola, P., Heitzeg, M. M., et al. (2011). Executive function in chronic pain patients and healthy controls: Different cortical activation during response inhibition in fibromyalgia. Journal of Pain, 12(12), 1219–1229. 10.1016/j.jpain.2011.06.007. 21945593 PMC3715316

[ref28] Hadi, M. A., McHugh, G. A., & Closs, S. J. (2019). Impact of chronic pain on patients’ quality of life: A comparative mixed-methods study. Journal of Patient Experience, 6(2), 133–141. 10.1177/2374373518786013. 31218259 PMC6558939

[ref29] Hart, R. P., Martelli, M. F., & Zasler, N. D. (2000). Chronic pain and neuropsychological functioning. Neuropsychology Review, 10(3), 131–149. 10.1023/A:1009020914358. 10983898

[ref30] Howard, F. M., El-Minawi, A. M., & Sanchez, R. A. (2000). Conscious pain mapping by laparoscopy in women with chronic pelvic pain. Obstetrics and Gynecology, 96(6), 934–939. 10.1016/S0029-7844(00)01056-5. 11084181

[ref31] Japee, S., Holiday, K., Satyshur, M. D., Mukai, I., & Ungerleider, L. G. (2015). A role of right middle frontal gyrus in reorienting of attention: A case study. Frontiers in Systems Neuroscience, 9, 23. 10.3389/fnsys.2015.00023. 25784862 PMC4347607

[ref32] Jones, G., Jenkinson, C., & Kennedy, S. (2004). The impact of endometriosis upon quality of life: A qualitative analysis. Journal of Psychosomatic Obstetrics and Gynecology, 25(2), 123–133. 10.1080/01674820400002279. 15715035

[ref33] Jones, G., Kennedy, S., Barnard, A., Wong, J., & Jenkinson, C. (2001). Development of an endometriosis quality-of-life instrument: The Endometriosis Health Profile-30. Obstetrics and Gynecology, 98(2), 258–264. 10.1016/S0029-7844(01)01433-8. 11506842

[ref34] Karimi, M., & Brazier, J. (2016). Health, health-related quality of life, and quality of life: What is the difference? PharmacoEconomics, 34(7), 645–649. 10.1007/s40273-016-0389-9. 26892973

[ref35] Khera, T., & Rangasamy, V. (2021). Cognition and pain: A review. Frontiers in Psychology, 12, 673962. 10.3389/fpsyg.2021.673962. 34093370 PMC8175647

[ref36] Koechlin, E., & Summerfield, C. (2007). An information theoretical approach to prefrontal executive function. Trends in Cognitive Sciences, 11(6), 229–235. 10.1016/j.tics.2007.04.005. 17475536

[ref37] Kumar, A., Gupta, V., & Maurya, A. (2010). Mental health and quality of life of chronic pelvic pain and endometriosis patients. SIS Journal of Projective Psychology and Mental Health, 17(2), 153–157. https://search.ebscohost.com/login.aspx?direct=true&db=a9h&AN=57572333&site=ehost-live&scope=site.

[ref38] Lamvu, G., Carrillo, J., Ouyang, C., & Rapkin, A. (2021). Chronic pelvic pain in women: A review. JAMA, 325(23), 2381–2391. 10.1001/jama.2021.2631. 34128995

[ref39] Landrø, N. I., Stiles, T. C., & Sletvold, H. (1997). Memory functioning in patients with primary fibromyalgia and major depression and healthy controls. Journal of Psychosomatic Research, 42(3), 297–306. 10.1016/S0022-3999(96)00301-7. 9130186

[ref40] Latthe, P., Latthe, M., Say, L., Gülmezoglu, M., & Khan, K. S. (2006). WHO systematic review of prevalence of chronic pelvic pain: A neglected reproductive health morbidity. BMC Public Health, 6(1), 1–7. 10.1186/1471-2458-6-177. 16824213 PMC1550236

[ref41] Li, R., Kreher, D. A., Gubbels, A. L., Palermo, T. M., Benjamin, A. R., Irvine, C. S., et al. (2023). Dysmenorrhea catastrophizing and functional impairment in female pelvic pain. Frontiers in Pain Research, 3, 1053026. 10.3389/fpain.2022.1053026. 36688085 PMC9853896

[ref42] Li, R., Li, B., Kreher, D. A., Benjamin, A. R., Gubbels, A., & Smith, S. M. (2020). Association between dysmenorrhea and chronic pain: A systematic review and meta-analysis of population-based studies. American Journal of Obstetrics and Gynecology, 223(3), 350–371. 10.1016/j.ajog.2020.03.002. 32151612

[ref43] Lopez-Garcia, P., Lesh, T. A., Salo, T., Barch, D. M., MacDonald, A. W., Gold, J. M., et al. (2016). The neural circuitry supporting goal maintenance during cognitive control: A comparison of expectancy AX-CPT and dot probe expectancy paradigms. Cognitive, Affective, & Behavioral Neuroscience, 16(1), 164–175. 10.3758/s13415-015-0384-1. PMC4819423 26494483

[ref44] MacGregor, B., Allaire, C., Bedaiwy, M. A., Yong, P. J., & Bougie, O. (2023). Disease burden of dysmenorrhea: Impact on life course potential. International Journal of Women’s Health, Volume 15, 499–509. 10.2147/ijwh.S380006. PMC10081671 37033122

[ref45] Maddern, J., Grundy, L., Castro, J., & Brierley, S. M. (2020). Pain in endometriosis. Frontiers in Cellular Neuroscience, 14, 590823. 10.3389/fncel.2020.590823. 33132854 PMC7573391

[ref46] Markham, R., Luscombe, G. M., Manconi, F., & Fraser, I. S. (2019). A detailed profile of pain in severe endometriosis. Journal of Endometriosis and Pelvic Pain Disorders, 11(2), 85–94. 10.1177/2284026519838948.

[ref47] Martucci, K. T., Shirer, W. R., Bagarinao, E., Johnson, K. A., Farmer, M. A., Labus, J. S., et al. (2015). The posterior medial cortex in urologic chronic pelvic pain syndrome: Detachment from default mode network. A resting-state study from the MAPP research network. Pain, 156(9), 1755. 10.1097/j.pain.0000000000000238. 26010458 PMC4545714

[ref48] Maulitz, L., Stickeler, E., Stickel, S., Habel, U., Tchaikovski, S., & Chechko, N. (2022). Endometriosis, psychiatric comorbidities and neuroimaging: Estimating the odds of an endometriosis brain. Frontiers in Neuroendocrinology, 65, 100988. 10.1016/j.yfrne.2022.100988. 35202605

[ref49] Melzack, R. (2001). Pain and the neuromatrix in the brain. Journal of Dental Education, 65(12), 1378–1382. 10.1002/j.0022-0337.2001.65.12.tb03497.x. 11780656

[ref50] Melzack, R., & Wall, P. D. (1965). Pain mechanisms: A new theory: A gate control system modulates sensory input from the skin before it evokes pain perception and response. Science, 150(3699), 971–979. 10.1126/science.150.3699.971. 5320816

[ref51] Moher, D., Shamseer, L., Clarke, M., Ghersi, D., Liberati, A., Petticrew, M., et al. (2015). Preferred reporting items for systematic review and meta-analysis protocols (PRISMA-P) 2015 statement. Systematic Reviews, 4(1), 1–9. 10.1186/2046-4053-4-1. 25554246 PMC4320440

[ref52] Moriarty, O., McGuire, B. E., & Finn, D. P. (2011). The effect of pain on cognitive function: A review of clinical and preclinical research. Progress in Neurobiology, 93(3), 385–404. 10.1016/j.pneurobio.2011.01.002. 21216272

[ref53] Morotti, M., Vincent, K., & Becker, C. M. (2017). Mechanisms of pain in endometriosis. European Journal of Obstetrics & Gynecology and Reproductive Biology, 209, 8–13. 10.1016/j.ejogrb.2016.07.497. 27522645

[ref54] Moulton, E. A., Schmahmann, J. D., Becerra, L., & Borsook, D. (2010). The cerebellum and pain: Passive integrator or active participator? Brain Research Reviews, 65(1), 14–27. 10.1016/j.brainresrev.2010.05.005. 20553761 PMC2943015

[ref55] Naliboff, B. D., Stephens, A. J., Afari, N., Lai, H., Krieger, J. N., Hong, B., et al. (2015). Widespread psychosocial difficulties in men and women with urologic chronic pelvic pain syndromes: Case–control findings from the multidisciplinary approach to the study of chronic pelvic pain research network. Urology, 85(6), 1319–1327. 10.1016/j.urology.2015.02.047. 26099876 PMC4479402

[ref56] Onitsuka, T., Shenton, M. E., Salisbury, D. F., Dickey, C. C., Kasai, K., Toner, S. K., et al. (2004). Middle and inferior temporal gyrus gray matter volume abnormalities in chronic schizophrenia: An MRI study. American Journal of Psychiatry, 161(9), 1603–1611. 10.1176/appi.ajp.161.9.1603. 15337650 PMC2793337

[ref57] Pani, P., Di Bello, F., Brunamonti, E., D’Andrea, V., Papazachariadis, O., & Ferraina, S. (2014). Alpha-and beta-band oscillations subserve different processes in reactive control of limb movements. Frontiers in Behavioral Neuroscience, 8, 383. 10.3389/fnbeh.2014.00383. 25414649 PMC4220745

[ref58] Peters, M. L. (2015). Emotional and cognitive influences on pain experience. Modern Trends in Pharmacopsychiatry, 30, 138–152. 10.1159/000435938. 26436897

[ref59] Phelps, C. E., Navratilova, E., & Porreca, F. (2021). Cognition in the chronic pain experience: Preclinical insights. Trends in Cognitive Sciences, 25(5), 365–376. 10.1016/j.tics.2021.01.001. 33509733 PMC8035230

[ref60] Povedano, M., Gascón, J., Gálvez, R., Ruiz, M., & Rejas, J. (2007). Cognitive function impairment in patients with neuropathic pain under standard conditions of care. Journal of Pain and Symptom Management, 33(1), 78–89. 10.1016/j.jpainsymman.2006.07.012. 17196909

[ref61] Price, D. D., Verne, G. N., & Schwartz, J. M. (2006). Plasticity in brain processing and modulation of pain. Progress in Brain Research, 157, 333–405. 10.1016/S0079-6123(06)57020-7. 17167920

[ref62] Qin, P., Wu, X., Huang, Z., Duncan, N. W., Tang, W., Wolff, A., et al. (2015). How are different neural networks related to consciousness? Annals of Neurology, 78(4), 594–605. 10.1002/ana.24479. 26290126

[ref63] Rezvanfard, M., Golesorkhi, M., Ghasemian-Shirvan, E., Safaei, H., Eghbali, A. N., Alizadeh, H., et al. (2016). Evaluation of inhibition response behavior using the go/no-go paradigm in normal individuals: Effects of variations in the task design. Acta Neuropsychologica, 14(4), 357–366. 10.5604/17307503.1227530.

[ref64] Rogers, P. A., Adamson, G. D., Al-Jefout, M., Becker, C. M., D’Hooghe, T. M., Dunselman, G. A., et al. (2017). Research priorities for endometriosis: Recommendations from a global consortium of investigators in endometriosis. Reproductive Sciences, 24(2), 202–226. 10.1177/1933719116654991. 27368878 PMC5933154

[ref65] Roth, R. S., Geisser, M. E., Theisen-Goodvich, M., & Dixon, P. J. (2005). Cognitive complaints are associated with depression, fatigue, female sex, and pain catastrophizing in patients with chronic pain. Archives of Physical Medicine and Rehabilitation, 86(6), 1147–1154. 10.1016/j.apmr.2004.10.041. 15954053

[ref66] Saunders, P. T., & Horne, A. W. (2021). Endometriosis: Etiology, pathobiology, and therapeutic prospects. Cell, 184(11), 2807–2824. 10.1016/j.cell.2021.04.041. 34048704

[ref67] Schimmelpfennig, J., Topczewski, J., Zajkowski, W., & Jankowiak-Siuda, K. (2023). The role of the salience network in cognitive and affective deficits. Frontiers in Human Neuroscience, 17, Article 1133367. 10.3389/fnhum.2023.1133367. PMC10067884 37020493

[ref68] Seminowicz, D. A., & Davis, K. D. (2007). Interactions of pain intensity and cognitive load: The brain stays on task. Cerebral Cortex, 17(6), 1412–1422. 10.1093/cercor/bhl052. 16908493

[ref69] Seminowicz, D. A., Wideman, T. H., Naso, L., Hatami-Khoroushahi, Z., Fallatah, S., Ware, M. A., et al. (2011). Effective treatment of chronic low back pain in humans reverses abnormal brain anatomy and function. Journal of Neuroscience, 31(20), 7540–7550. 10.1523/jneurosci.5280-10.2011. 21593339 PMC6622603

[ref70] Seo, J., Kim, S.-H., Kim, Y.-T., Song, H.-J., Lee, J.-J., Kim, S.-H., et al. (2012). Working memory impairment in fibromyalgia patients associated with altered frontoparietal memory network. PLoS One, 7(6), e37808. 10.1371/journal.pone.0037808. 22715371 PMC3370998

[ref71] Sepulcri, R. d. P., & do Amaral, V. F. (2009). Depressive symptoms, anxiety, and quality of life in women with pelvic endometriosis. European Journal of Obstetrics & Gynecology and Reproductive Biology, 142(1), 53–56. 10.1016/j.ejogrb.2008.09.003. 19010584

[ref72] Serrano, P. V., Zortea, M., Alves, R. L., Beltran, G., Deliberali, C. B., Maule, A., et al. (2022). Association between descending pain modulatory system and cognitive impairment in fibromyalgia: A cross-sectional exploratory study. Frontiers in Behavioral Neuroscience, 16, Article 917554. 10.3389/fnbeh.2022.917554. 36248031 PMC9559397

[ref73] Shen, Z., Yu, S., Wang, M., She, T., Yang, Y., Wang, Y., et al. (2019). Abnormal amygdala resting-state functional connectivity in primary dysmenorrhea. Neuroreport, 30(5), 363–368. 10.1097/WNR.0000000000001208. 30762615

[ref74] Smith, M. T., Jr., Remeniuk, B., Finan, P. H., Speed, T. J., Tompkins, D. A., Robinson, M., et al. (2019). Sex differences in measures of central sensitization and pain sensitivity to experimental sleep disruption: Implications for sex differences in chronic pain. Sleep, 42(2), zsy209. 10.1093/sleep/zsy209. 30371854 PMC6369729

[ref75] Sperschneider, M. L., Hengartner, M. P., Kohl-Schwartz, A., Geraedts, K., Rauchfuss, M., Woelfler, M. M., et al. (2019). Does endometriosis affect professional life? A matched case-control study in Switzerland, Germany and Austria. BMJ Open, 9(1), e019570. 10.1136/bmjopen-2017-019570. PMC6340011 30782670

[ref76] Steiner, G. Z., Barry, R. J., Wassink, K., De Blasio, F. M., Fogarty, J. S., Cave, A. E., et al. (2020). Neuronal correlates of cognitive control are altered in women with endometriosis and chronic pelvic pain. Frontiers in Systems Neuroscience, 14, 593581. 10.3389/fnsys.2020.593581. 33390910 PMC7772245

[ref77] Stratton, P., & Berkley, K. J. (2011). Chronic pelvic pain and endometriosis: Translational evidence of the relationship and implications. Human Reproduction Update, 17(3), 327–346. 10.1093/humupd/dmq050. 21106492 PMC3072022

[ref78] Szabo, E., Timmers, I., Borsook, D., Simons, L. E., & Sieberg, C. B. (2022). Altered anterior insula functional connectivity in adolescent and young women with endometriosis-associated pain: Pilot resting-state fMRI study. European Journal of Paediatric Neurology, 41, 80–90. 10.1016/j.ejpn.2022.10.004. 36375399 PMC9722632

[ref79] Tejera-Alonso, A., Fernández-Palacios, F. G., Pacho-Hernández, J. C., Naeimi, A., de-la Llave-Rincón, A. I., Ambite-Quesada, S., et al. (2024). Effects of executive functions and cognitive variables in experimentally induced acute pain perception during a distraction task: A study on asymptomatic pain-free individuals. Life, 14(9), 1141. 10.3390/life14091141. 39337924 PMC11433093

[ref80] Tripp, D. A., Curtis Nickel, J., Landis, J. R., Wang, Y. L., Knauss, J. S., & Group, C. S (2004). Predictors of quality of life and pain in chronic prostatitis/chronic pelvic pain syndrome: Findings from the National Institutes of Health chronic prostatitis cohort study. BJU International, 94(9), 1279–1282. 10.1111/j.1464-410X.2004.05157.x. 15610105

[ref81] Uddin, L. Q. (2016). Salience network of the human brain. Elsevier Science, Academic press.

[ref82] Uddin, L. Q., Clare Kelly, A., Biswal, B. B., Xavier Castellanos, F., & Milham, M. P. (2009). Functional connectivity of default mode network components: Correlation, anticorrelation, and causality. Human Brain Mapping, 30(2), 625–637. 10.1002/hbm.20531. 18219617 PMC3654104

[ref83] Verdejo-García, A., López-Torrecillas, F., Calandre, E. P., Delgado-Rodríguez, A., & Bechara, A. (2009). Executive function and decision-making in women with fibromyalgia. Archives of Clinical Neuropsychology, 24(1), 113–122. 10.1093/arclin/acp014. 19395361

[ref84] Ware, J. E., Jr., & Sherbourne, C. D. (1992). The MOS 36-item short-form health survey (SF-36): I. Conceptual framework and item selection. Medical Care, 30(6), 473–483. https://www.jstor.org/stable/3765916. 10.1097/00005650-199206000-00002. 1593914

[ref85] Yong, P. J., Williams, C., Bedaiwy, M. A., & Allaire, C. (2020). A proposed platform for phenotyping endometriosis-associated pain: Unifying peripheral and central pain mechanisms. Current Obstetrics and Gynecology Reports, 9(3), 89–97. 10.1007/s13669-020-00288-8.

